# Importin 13 promotes NSCLC progression by mediating RFPL3 nuclear translocation and hTERT expression upregulation

**DOI:** 10.1038/s41419-020-03101-9

**Published:** 2020-10-20

**Authors:** Bisan Abdalfatah Zohud, Ping Guo, Batoul Abdalfatah Zohud, Fengzhou Li, Jiao J. Hao, Xiu Shan, Wendan Yu, Wei Guo, Yu Qin, Xin Cai

**Affiliations:** 1 The First Affiliated Hospital of Dalian Medical University, 116011 Dalian, China; 2 Institute of Cancer Stem Cell, Dalian Medical University, 116044 Dalian, China; 3 The First Affiliated Hospital of Nanchang University, Nanchang, China

**Keywords:** Non-small-cell lung cancer, Tumour biomarkers

## Abstract

Our previous studies have reported that RFPL3 protein exerts its unique function as a transcriptional factor of hTERT promoter after being transported into the lung cancer cell nucleus. However, the detailed mechanism by which RFPL3 undergoes nuclear transport has not been reported yet. Here, we identified RFPL3 as a potential import cargo for IPO13, which was found to be overexpressed in NSCLC cells and tissues. IPO13 interacted with RFPL3 in lung cancer cells, and the knockdown of IPO13 led to the cytoplasmic accumulation of RFPL3, the decreased anchoring of RFPL3 at hTERT promoter, and the downregulation of hTERT expression. Moreover, IPO13 silencing suppressed tumor growth in vitro and in vivo. IHC analysis confirmed the positive correlation between the expression levels of IPO13 and hTERT in the tumor tissues from patients with lung cancer. Furthermore, the mechanistic study revealed that IPO13 recognized RFPL3 via a functional nuclear localization signal (NLS), which is located in the B30.2 domain at the C-terminal region of RFPL3. Of note, the presence of EGFR mutations was significantly related to the increased IPO13 expression. The EGFR-TKI Osimertinib downregulated IPO13 expression level in NSCLC cell lines with EGFR mutations, but not in EGFR wild-type ones. In summary, our data suggest that inhibition of IPO13 transport activity itself might be an alternative and potential therapeutic strategy for NSCLC.

## Introduction

In the eukaryotic cell, macromolecules trafficking between the nucleus and cytoplasm are crucial for many cellular processes. The nucleocytoplasmic transport process occurs across the only entrance across the nuclear envelope, nuclear pore complex (NPC). NPC is built of approximately 30 different kinds of nucleoporins (Nup)^1,2^. The most active transport processes are intermediated by karyopherins, soluble nuclear protein transport receptors (NTRs) that recognize their cargoes directly through a specific amino acid sequence and facilitate transporting across the NPC. It has been classified into the importin and exportin based on the transport direction^3^. Importins recognize their cargo proteins via nuclear localization signal (NLS) characterized by amino acids rich in arginine and lysine with a positive charge, which was further classified into monopartite and bipartite^4^. Ran GTPase controls association and dissociation of NTR-cargo complexes, which determines the transport direction. In the cytoplasm, importins bind their protein cargoes via NLSs in the absence of RanGTP and release them in the nucleus when binding RanGTP. In contrast, exportins form a trimeric complex with their cargoes in the nucleus in the presence of RanGTP. Then, in the cytoplasm, these complexes are dissociated upon RanGTP hydrolysis^5–7^. The RanGTP hydrolysis constitutes a significant source of metabolic energy in import–export transport cycles. NTRs include both karyopherin-alpha (KPNA) and karyopherin-beta (KPNB) family. KPNB subunits mediate the docking of KPNA-cargo complex to the NPC, thereby facilitating the nuclear import of cargo proteins. Meanwhile, KPNB subunits can directly interact with the target cargo^8^. Recently, several reports have proposed karyopherins as novel targets of the anticancer strategy. KPNA4, encoding karyopherin-α4, is overexpressed in head and neck of squamous cell carcinoma, and promotes malignant phenotypes by sustaining Ras/ERK pathway signaling^9^. Targeting karyopherin-beta1 (KPNB1) can significantly suppress prostate cancer progression by blocking the nuclear import of oncogenic transcription factor NF-κB and c-Myc^10^. Furthermore, inhibiting KPNB1’s nuclear transport function suppressed PD-L1 expression level in irradiated human head and neck squamous cell carcinoma (HNSCC) cells^8^. As one of the KPNB subtypes, Importin 13 (IPO13) is a bidirectional transporter protein with highly flexible structure that intermediates the nuclear import of some cargoes (Mago-Y14 and Ubc9) as well as the export of some others (eIF1A and HMG20A)^11,12^. IPO13 highly expresses in various tissues, including heart^13^, cornea^14^, fetal lung^15–17^, brain^18^, and endometrial carcinoma^19^. However, the expression level and the potential function of IPO13 in various cancers are still unknown.

Human telomerase reverse transcriptase (hTERT), which maintains telomere length by adding TTAGGG sequence repeats to the ends of telomere, shows overexpression in 85–90% human cancers^20,21^, and plays a critical role in cancer cell proliferation^22^. Therefore, hTERT upregulation has been considered as an accessible tumor biomarker and a novel target for anticancer therapeutics^20^. In various cancers, it is known that the high expression of hTERT is regulated at the transcriptional level. However, the precise regulatory mechanisms of hTERT expression in lung cancer are still poorly defined. Hence, identification of the specific transcriptional factors and the revelation of their nuclear transport mechanisms would enable to reduce hTERT expression and ultimately inhibit tumor growth^23^. Our team previously discovered that RFPL3 is an essential hTERT promoter-binding protein that controls hTERT expression and activity in lung cancer^24^.

RFPL3 is a member of the Ret Finger Protein-Like (RFPL) family that plays a regulatory role in human embryonic development and neurogenesis^25,26^. RFPL3 stimulates the integration activity of HIV-1 virus pre-integration complex (PIC) in vitro^27^. Also, RFPL3’s gene location close to telomeres facilitates its function as an hTERT promoter-binding protein^26^, which plays a vital role in regulating hTERT transcription and promoting its expression, therefore increasing telomerase activity and enhancing proliferation of lung cancer cells. Additionally, RFPL3 overexpression was correlated positively with short overall survival (OS) and nodal metastasis in lung adenocarcinoma patients^24^. In NSCLC, the overexpression of RFPL3 protein and its nuclear localization are significant markers of tumorigenesis and development. However, the mechanism by which RFPL3 is transported from the cytoplasm to the nucleus has not been reported yet.

Presently, in our study, we aimed to identify the molecular mechanism that mediates the nuclear translocation of RFPL3 in lung cancer. We observed for the first time that IPO13 mediated the nuclear import of RFPL3 through a functional NLS within RFPL3 and subsequent hTERT expression upregulation. Besides, IPO13 was found to play a critical role in NSCLC progression, which was implied by the proliferation promotion of NSCLC cells in vitro and the influence on tumor growth of an NSCLC cancer model in vivo. Further mechanistic studies showed that EGFR signaling might regulate the expression of IPO13 in NSCLC with EGFR mutations. These results illuminate that IPO13 might upregulate hTERT expression and promote cancer cell viability via mediating RFPL3 nuclear transport mechanism. Therefore, IPO13 should be suggested as a novel target for NSCLC therapies.

## Materials and methods

### Cell culture

Both human lung adenocarcinoma cells (H1299, A549, and H1975) and human normal epithelial lung cells (HBE) were purchased from ATCC (Manassas, VA). H1299 and HBE grew in Dulbecco’s modified Eagle medium (DMEM) containing 10% fetal bovine serum (FBS). While A549 and H1975 were cultured in RPMI-1640 medium (Invitrogen) supplemented with 10% heat-inactivated FBS. All mentioned cells were maintained at 37 °C in a moist incubator containing 5% CO_2_.

### Western blot assay

Protein from cultured cells or tissues lysates was quantified by BCA assay kit and loaded onto 12% or 10% SDS-PAGE, then transferred onto the PVDF membrane electrophoretically. To block the membranes, 7% nonfat milk in TBST was used for 2 h at room temperature (RT). The desired protein bands immunoblotted with a primary antibody against β-actin (cat: 8480T, Cell Signaling Technology), RFPL3 (cat: ab-128090, Abcam), (cat: sc-133955, SANTA CRUZ), Flag (cat: 66008-2-Ig, proteintech), IPO13 (NBP1-31508, Novus Biotechnology), Lamin B (cat: 12987-1-AP, Proteintech), and hTERT (cat: sc7212, Santa Cruz) overnight at 4 °C. After washing with TBST buffer three times, the secondary antibody was incubated at RT for 2 h. Finally, immunoreactive bands were visualized using a chemiluminescence kit (ECL). Image J software was used to quantify the intensities of various protein bands.

### Knockdown of IPO13 (siRNA and shRNA)

To inhibit IPO13 expression, short-hairpin RNA (shRNA) was synthesized by GeneCopoeia (USA) and cloned into a psi-LVRU6P vector. NSCLC cells were transfected with sh-IPO13 RNA (5′-TAATACGACTCACTATAGGG-3′; 5′-CTGGAATAGCTCAGAGGC-3′). We used A549 cells to establish a sh-IPO13 stable cell line. Meanwhile, two siRNAs target IPO13 synthesized by GenePharma (China). The siRNA-1 sequence is 5′-CCCAGGAUGUGCUGAUGAATT-3′; 5′-UUCAUCAGCACAUCCUGGGTT-3′, and siRNA-2 sequence is 5′-CCAGGGAUCAUCCUGAUAUTT-3′; 5′-AUAUCAGGAUGAUCCCUGGTT-3′. Negative control siRNA sequence is 5′-GCACUCGUCAACAUGAUUATT-3′; 5′-UAAUCAUGUUGACGAGUGCTT-3′. We plated the desired cells in a 96-well plate (1000 cells/well) or a six-well plate and transfected with siRNA plasmids using Lipofectamine 3000 (Invitrogen). Twenty-four or forty-eight hours later, cell viability, protein expression, and immunofluorescence were performed.

### RFPL3 plasmids

The RFPL3 full-length (FL) and truncation-mutation plasmids with FLAG tag in pcDNA3.1 vector were purchased from YOU Biotechnology (China). Then A549 cells were transfected with plasmids using Lipofectamine 3000 reagent.

### Immunofluorescence localization and laser confocal microscopy

Cells were placed on clean glass coverslip in a six-well plate for 24–48 h. Cells were immobilized with 4% paraformaldehyde (PFA) for 30 min, washed with PBS three times, and then 0.2% Triton X-100 (formulated in PBS) was added to permeate cells for 5 min. Cells were washed and blocked with 10% bovine serum albumin (BSA) in PBS solution. The RFPL3, IPO13, or FLAG primary antibodies were diluted with 1% BSA (1:200) and incubated overnight at 4 °C. After PBS washing three times, cells were incubated for 1 h with fluorescein isothiocyanate-conjugated and TRITC-conjugated secondary antibodies in a humidified chamber in darkness at room temperature. Nuclei of stained cells were mounted with DAPI (1:10,000) for 2 min, followed by several PBS washes. The protein’s localization was visualized using a (Leica DM 14000B) confocal fluorescence microscope.

### Co-immunoprecipitation (Co-IP)

The protein lysates obtained from different cells were incubated with a polyclonal antibody against IPO13 or rabbit IgG on the rotator, overnight at 4 °C. Then agarose-coupled protein A/G beads (Santa Cruz Biotechnology) were incubated with proteins for 6 h at 4 °C. After washing with PBSI (PBS + protease inhibitors) four times, we resuspended the precipitates in 40 μl of 4× loading buffer and boiled at 95 °C for 5–10 min. The proteins were subjected to western blot analysis.

### Cell viability

Lung cancer cells were seeded into 96-well plates (1000 cells per well). At 50% confluence, we treated cells with different concentrations of Osimertinib (AstraZeneca) or transfected with siRNAs. Forty-eight hours later, 10% MTT reagent diluted in the media was added and incubated for 3–4 h. After MTT was discarded, 100 μl of DMSO per well was added. Finally, cell viability was measured at 490-nm wavelength.

### Animal studies

Animal experiments were permitted by the Animals Research Ethics Committee of Dalian Medical University. The BALB/C male nude mice aged 4–6 weeks were acquired from Beijing Vital River Laboratory Animal Technology Co. Mice were subcutaneously injected with sh-IPO13 or NC-IPO13 A549 cells (5 × 10^6^ cells/100 μl) into their right flank (*n* = 5/group). The xenograft tumor growth was recorded by digital caliper on the specified days. The formula calculating the tumor volume is vol = (length × width^2^)/2. Thirty days after implantation, we sacrificed the mice; then tumor weight was recorded. Part of the tissues was transferred immediately to liquid nitrogen to use later for western blot; another part was fixed in 4% PFA for IHC staining.

### Immunohistochemical staining assay

NSCLC specimens included in this study were collected from patients being informed. The study was permitted by the Medical Ethical Committee of the First Affiliated Hospital, Dalian Medical University. Briefly, dissected tissues were fixed in 4% formalin overnight, washed with PBS, and embedded in paraffin. Then tumor tissue slides were deparaffinized with xylene, rehydrated with ethanol, and stained using SP-9000 Kit (ZSGB-BIO, China). The primary antibody against RFPL3, IPO13, or hTERT with (1:200) dilution ratio was incubated overnight at 4 °C in a humidified chamber. Hematoxylin was used to counterstain the nucleus.

### Pull-down assay

Binding of RFPL3 to hTERT promoter was assessed by pull-down assay. In total, 400 μg of A549 stable cell line nuclear extracts, 4 μg of biotin-labeled dsDNA probe of hTERT promoter (–378 to +60) where the primers were obtained from TAKARA Company, and 50 μl of streptavidin- conjugated agarose beads were incubated on a rotating shaker at 4 °C overnight. After centrifugation for 10 min, the precipitate was washed three times with 500 μl of PBSI (5 μl of PMSF, 40 μl of NaF, 20 μl of β-glycerophosphate-Na, and 2 ml of PBS). The protein–DNA–bead complex precipitate was resuspended in 50 μl of 2× loading buffer and then boiled at 100 °C for 5–10 min. Later, supernatant proteins were analyzed by western blot using an antibody against RFPL3.

### ChIP assay

Chromatin immunoprecipitation (ChIP) was performed as described before^28^. The primer sequences used for PCR reaction were as the following: 5′-ACCCTGGGAGCGCGAGCGGC-3′; 5′-GGGGCGGGGTCCGCGCGGAG-3′.

### Subcellular fractionation

The cytoplasmic fractionation was isolated using 500 μl of Cytoplasmic lysis buffer (10 mM KCl, 10 mM HEPES, 3 mM MgCl_2_.6H_2_O, 300 mM sucrose, and 10% NP-40) in addition to protease inhibitors (2 μl of Na_3_VO_4_, 5 μl of PMSF, 40 μl of NaF, 4 μl of leupeptin, 20 μl of β-glycerophosphate-Na, and 10 μl of DTT). The supernatant containing cytoplasmic proteins was collected after centrifugation for 10 min at 4 °C. Then we resuspended the pellet with 200 μl of nucleus lysis buffer with protease inhibitors (1.5 mM MgCl_2_.6H_2_O, 20 mM HEPES, 100 mM NaCl, 1 mM EDTA, and 10% glycerol) incubated on ice for 30 min and centrifuged at 14,000 × *g* for 30 min at 4 °C. Then supernatants were collected as nuclear proteins and kept at −80 °C for the next determination.

### Bioinformatics and gene-set enrichment analysis (GSEA)

GSEA was used to understand the biological functions of IPO13 and reveal Genes Ontology (GO) and Kyoto Encyclopedia of Gene and Genome (KEGG) that correlated to IPO13 expression. Data of 524 NSCLC cases that were used in GSEA came from NCBI Gene Expression Omnibus. Enrichment analysis was performed using gene sets with a false-discovery rate (FDR) < 0.25 and a nominal *P* value < 0.05.

### Statistical analysis

GraphPad Prism 5.0 v software was applied to analyze the experimental data and visualize the experimental results in the form of graphs. All the results were expressed as mean ± standard deviation (S.D) or individual data. The Pearson Chi-square (X^2^) test was used to compare the relation between IPO13/RFPL3 expression and clinicopathological variables of NSCLC patients. The *t* test and one-way ANOVA were used to determine statistical significance. Values of ^*^P < 0.05, ^**^P < 0.01, ^***^P < 0.001, ^****^P < 0.0001 were considered statistically significant.

## Results

### Upregulation of *IPO13* expression in *NSCLC* cells and tumor tissues

**Fig. 1:**
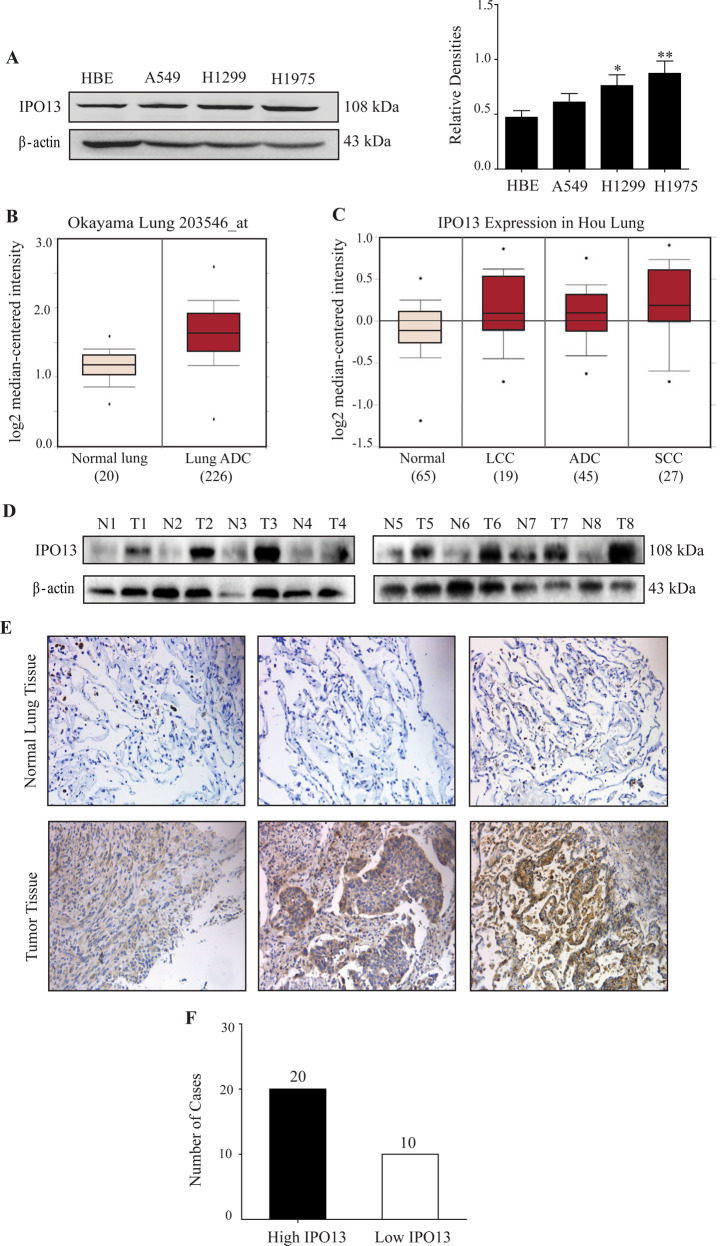
IPO13 was overexpressed in NSCLC cells and tumor tissues. **A** IPO13 protein levels were examined by western blot in normal epithelial lung cell HBE, and NSCLC cell lines (A549, H1299, and H1975); quantitation of relative IPO13 protein expression compared to HBE. The data were represented as mean ± S.D., Student’s *t* test (*n* = 3, *P < 0.05, **P < 0.01). **B** Analysis of IPO13 expression in lung cancer from Oncomine database. **C** Boxplots comparing IPO13 expression levels in the normal lung (65), large-cell lung carcinoma (19), lung adenocarcinoma (45), and squamous cell lung carcinoma (27) in 156 samples from Oncomine database. **D** The expression of IPO13 in lung cancer tissues and adjacent normal lung tissues from eight cases was detected by western blotting. **E** Immunohistochemical analysis of IPO13 expression in NSCLC tumor tissue and adjacent normal tissues (scale bar = 100 μm). **F** Based on IHC analysis, the number of cases with high and low levels of IPO13 are shown.

A nuclear transporter protein IPO13 was highly expressed in the lung epithelial cells, and is rarely studied in lung cancer. IPO13 protein levels were evaluated in three non-small-cell lung cancer cell lines compared with immortalized bronchial epithelial cells (HBE). Immunoblot results revealed that IPO13 expression in A549, H1299, and H1975 was higher than the normal cells (HBE). Meanwhile, the highest IPO13 protein level was detected in H1975 (*P* < 0.01) cell line that is harboring an active mutation in exon 21 (L858R) along with (T790M) secondary mutation in exon 20 (Fig. 1A). Furthermore, based on the Oncomine database, we found statically significant overexpression of IPO13 in lung adenocarcinoma compared with normal lung (Fig. 1B). Additionally, the IPO13 mRNA levels in 156 patient samples showed that the IPO13 expression was higher in large-cell lung carcinoma, squamous cell lung carcinoma, and lung adenocarcinoma than in normal tissues (Fig. 1C).

To assess the clinical significance of IPO13 in NSCLCs, western blot was performed to examine the IPO13 expression levels in tissue specimens collected from The First Affiliated Hospital of Dalian Medical University, including NSCLC and non-tumor lung tissues (*n* = 8). The immunoblot results confirmed that IPO13 expression in NSCLC tissues was significantly higher than that in normal tissues (Fig. 1D). Moreover, IPO13 in 30 fresh-tissue specimens from patients with NSCLC was examined by immunohistochemistry staining (IHC) (Fig. 1E). IPO13 is highly expressed in lung cancer tissue (high IPO13 in 20 cases, and low IPO13 in 10 cases) in comparison with adjacent normal tissues (Fig. 1F).

To further profile IPO13, we analyzed Oncomine data and performed RT-PCR to examine the expression levels of KNPB family members. The mRNA level of IPO13 was significantly higher than other KPNB members in NSCLC by comparison to the normal tissues or cell lines (Supplementary Fig. 1). Collectively, these findings identified IPO13 as a uniquely overexpressed KPNB subunit in NSCLC.

### Identification of RFPL3 as a potential cargo for IPO13

**Fig. 2:**
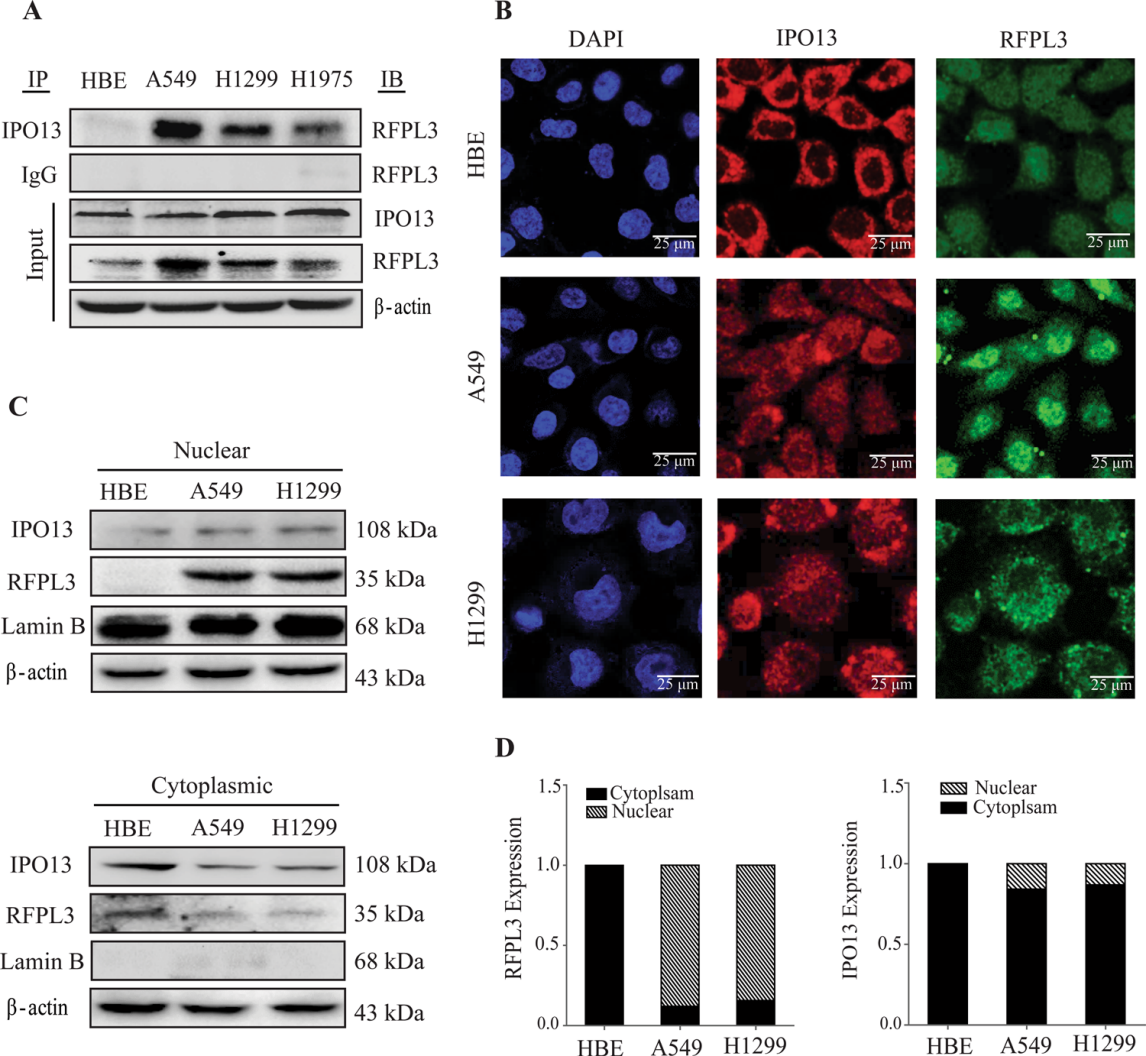
RFPL3 is an IPO13-cargo protein. **A** The protein interaction between RFPL3 and IPO13 has been detected in lung cancer cell lysates by Co-IP assay. **B** Co-localization and subcellular localization of RFPL3 and IPO13 in lung cancer and normal cells A549, H1299, and HBE was examined using confocal microscopy. Scale bar 25 μm. **C** IPO13 and RFPL3 expression levels in nuclear and cytoplasmic fractions were detected, respectively, by immunoblot assay. **D** Quantification of the relative protein expression compared to the control sample.

IPO13 belongs to the karyopherin-beta family, which mediates active nuclear transport of many cargoes. Regarding the finding that RFPL3 localized in the nucleus in lung cancer cells, we speculate that IPO13 might participate in the nucleocytoplasmic transport of RFPL3. To confirm whether RFPL3 is an IPO13-cargo protein, co-immunoprecipitation was initially performed (Fig. 2A). The IPO13 protein was immunoprecipitated with a human polyclonal anti-IPO13 antibody. Then western blot analysis was used to confirm the interaction by the anti-RFPL3 antibody. The results confirmed that RFPL3 coimmunoprecipitated with IPO13 in lung cancer cell lines, but no interaction was detected in HBE. To further confirm the hypothesis, the subcellular distribution of RFPL3 and IPO13 was evaluated by immunostaining and confocal imaging in numerous cell lines (Fig. 2B). Also, immunoblotting analysis of nuclear and cytoplasmic fractions was performed using Lamin B as a marker of nuclear protein (Fig. 2C). The analysis revealed that the nuclear RFPL3 portion was predominant in NSCLCs (A549 and H1299). Meanwhile, IPO13 mainly localized in the cytoplasmic fraction of HBE and in both subcellular fractions of A549 and H1299. The above results suggest that RFPL3 was a protein cargo of IPO13. IPO13/RFPL3 interaction might regulate RFPL3 nuclear import, which is a vital prerequisite for RFPL3 transcriptional regulatory functions.

### Knockdown of IPO13 prevents the nuclear entry of RFPL3

**Fig. 3:**
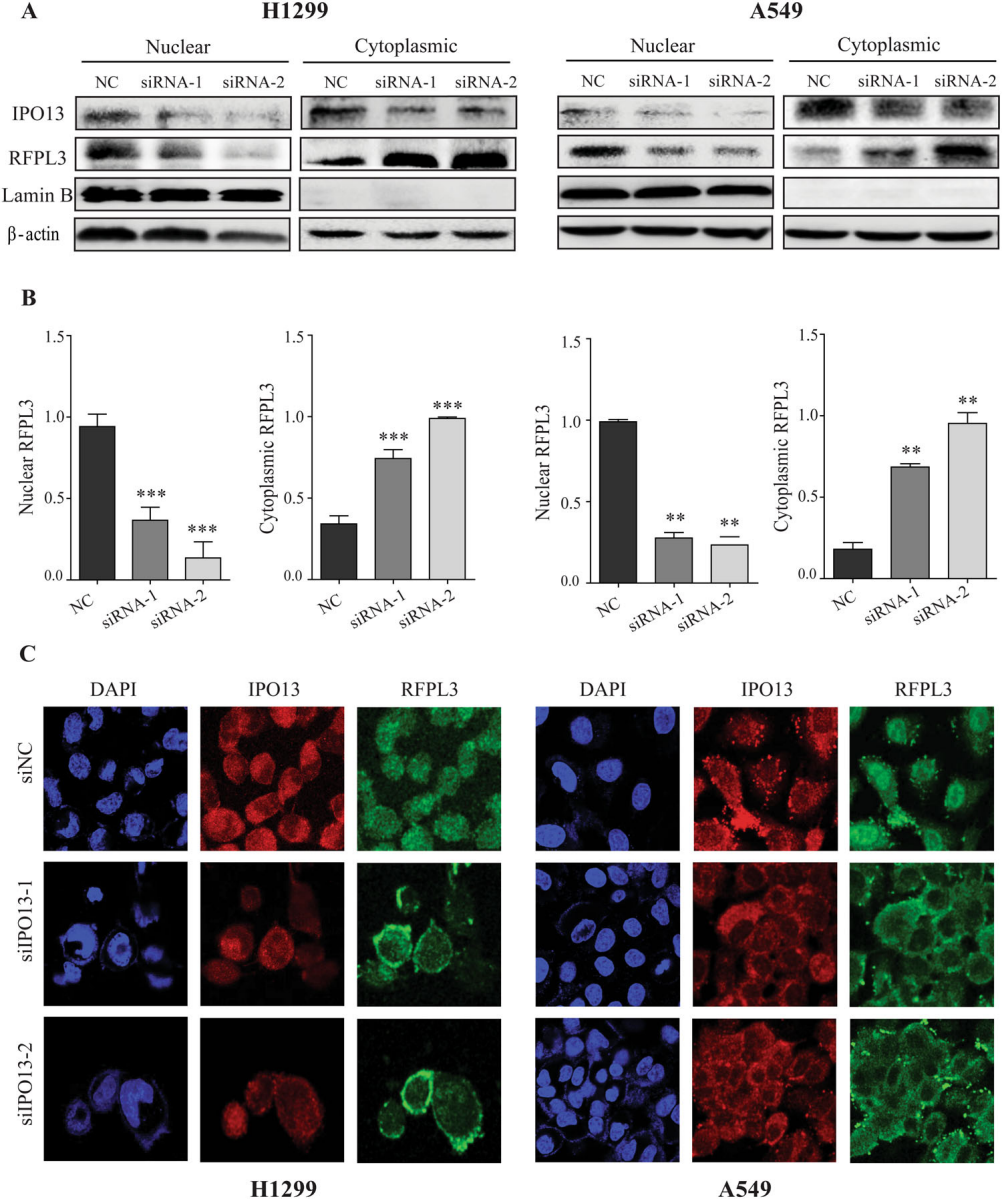
The nuclear import of RFPL3 mediated through IPO13. **A** Cells were transfected with siRNAs for 48 h to knock down IPO13. Immunoblot analysis shows the subcellular fractionation of RFPL3 or IPO13 proteins. **B** Histograms show densitometric analysis of nuclear and cytoplasmic RFPL3 from **A**. Student’s *t* test, **P < 0.01, ***P < 0.001. **C** H1299 and A549 cells were transfected with siRNAs for 48 h to knock down IPO13. Immunofluorescence staining (IF) was performed to show a substantial altered RFPL3 pattern after IPO13 depletion. Scale bar, 25 μm.

For further validation of the IPO13 role in RFPL3’s nuclear translocation, two different siRNAs targeting IPO13 were transfected to individually suppress the expression of IPO13 in two NSCLC cell lines H1299 and A549. In 48 h after the transient transfection of siRNAs, immunoblot analysis of cytoplasmic and nuclear proteins showed a cytoplasmic/membranous accumulation of RFPL3 in H1299 (*P* < 0.001) and A549 (*P* < 0.01) cells after downregulation of IPO13 (Fig. 3A, B), confirming that IPO13 can regulate the nuclear entry of RFPL3. As well, confocal imaging was performed after knockdown of IPO13 expression, which has also shown consistent results about RFPL3 subcellular localization (Fig. 3C). These findings strongly confirm that IPO13 functions as a nuclear import protein of RFPL3.

### Specific domain of RFPL3 is essential for its nuclear localization

**Fig. 4:**
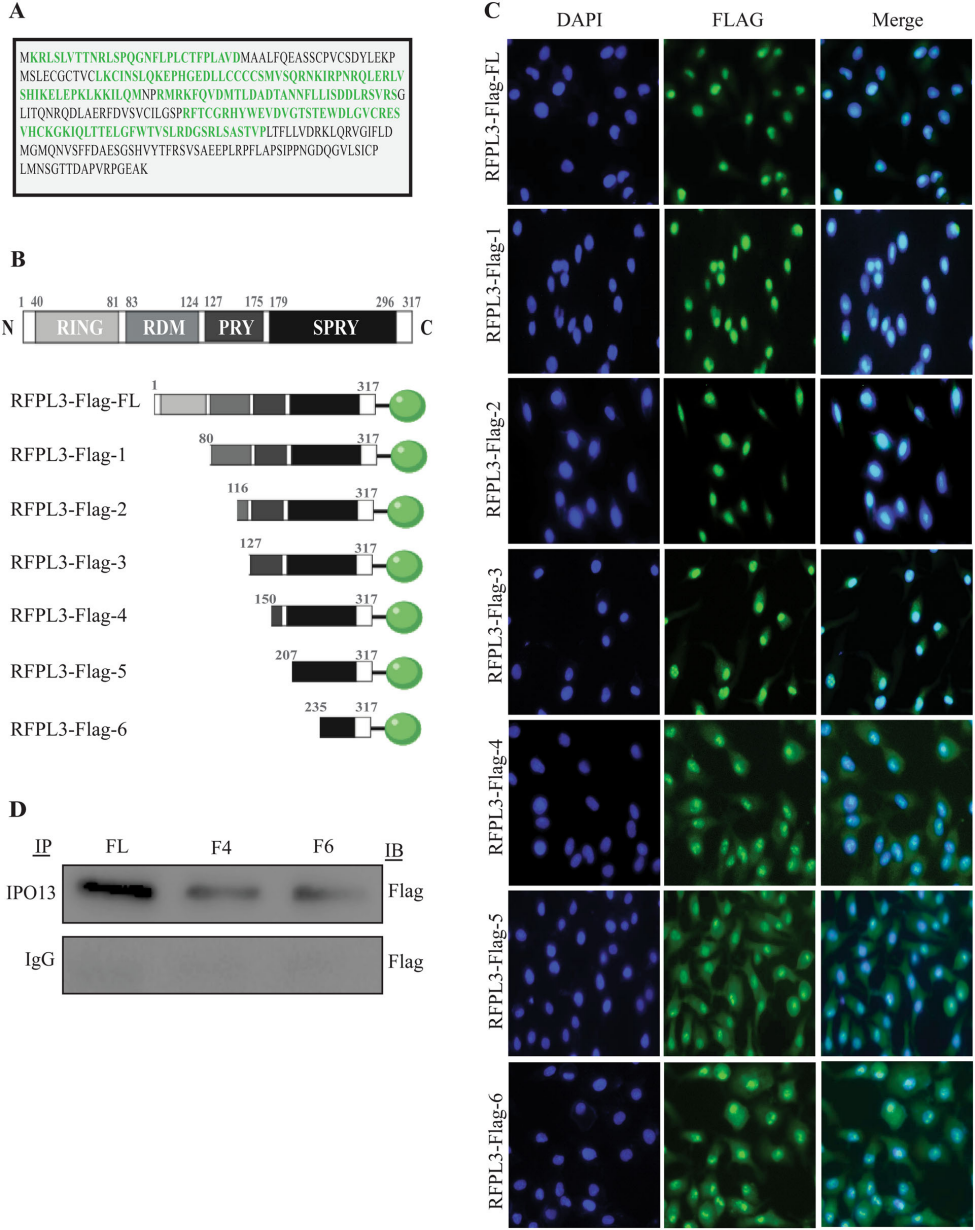
Mapping of nuclear localization signal sites in RFPL3. **A** The predicted nuclear localization sequences of RFPL3 using bioinformatics software cNLS Mapper. **B** Schematic of RFPL3 protein and its truncation mutants tagged with FLAG. **C** A549 cells transfected with RFPL3-FLAG-conjugated plasmids. Forty-eight hours later, cells stained with Flag antibody; then immunofluorescent assay was performed. Scale bar, 50 μm. **D** Flag-tagged RFPL3 and mutants were transiently transfected. Forty-eight hours post transfection, cells were lysed and immunoprecipitated with anti-IPO13 or rabbit IgG control and blotted with anti-Flag.

As reported, a human RFPL3 protein is composed of the N-terminal zinc-finger RING domain, and the C terminus contains a B30.2 domain that comprises SPRY and PRY motifs. Additionally, RDM, RFPL-defining motif, is flanked by the previous two domains (Fig. 4B). Nuclear localization signal (NLS), a particular amino acid sequence within RFPL3, regulates RFPL3 shuttling by binding to IPO13. To predict NLS motifs, we used the cNLS Mapper (http://nls-mapper.iab.keio.ac.jp), which revealed that RFPL3 has more than one bipartite or monopartite NLS (Fig. 4A). Based on this prediction, we constructed various FLAG-fused fragments of RFPL3 (truncation mutations and wild type) (Fig. 4B). Then, A549 cells were transfected with these plasmids. By 48 h after transfection, immunofluorescence microscopy was used to examine their subcellular localization (Fig. 4C). As predicted, full-length RFPL3-FLAG (1–317) localized in the nucleus. The deletion of the RING domain (80–317) did not affect RFPL3 nuclear localization. Further, truncation mutants of the RING and RDM domains were performed; the fluorescent pattern of F2 (116–317) and F3 (127–317) showed nuclear localization. Additional deletion of PRY and SPRY regions in RFPL3-Flag (150–317), (207–317), and (235–317) resulted in the distribution of RFPL3 throughout the cell with a preference to the cytoplasm. To further confirm the relevance of B30.2 domain with IPO13, we determined whether RFPL3 fragments can still bind endogenous IPO13. As IP results shown in Fig. 4D, RFPL3-Flag-4 (150–317) and RFPL3-Flag-6 (235–317) showed weak interaction with IPO13 compared to full-length RFPL3. These results indicate that SPY and SPRY regions located in the B30.2 domain at the C terminus are critical for RFPL3 nuclear localization.

### Depletion of IPO13 inhibits cell proliferation by hTERT downregulation

**Fig. 5:**
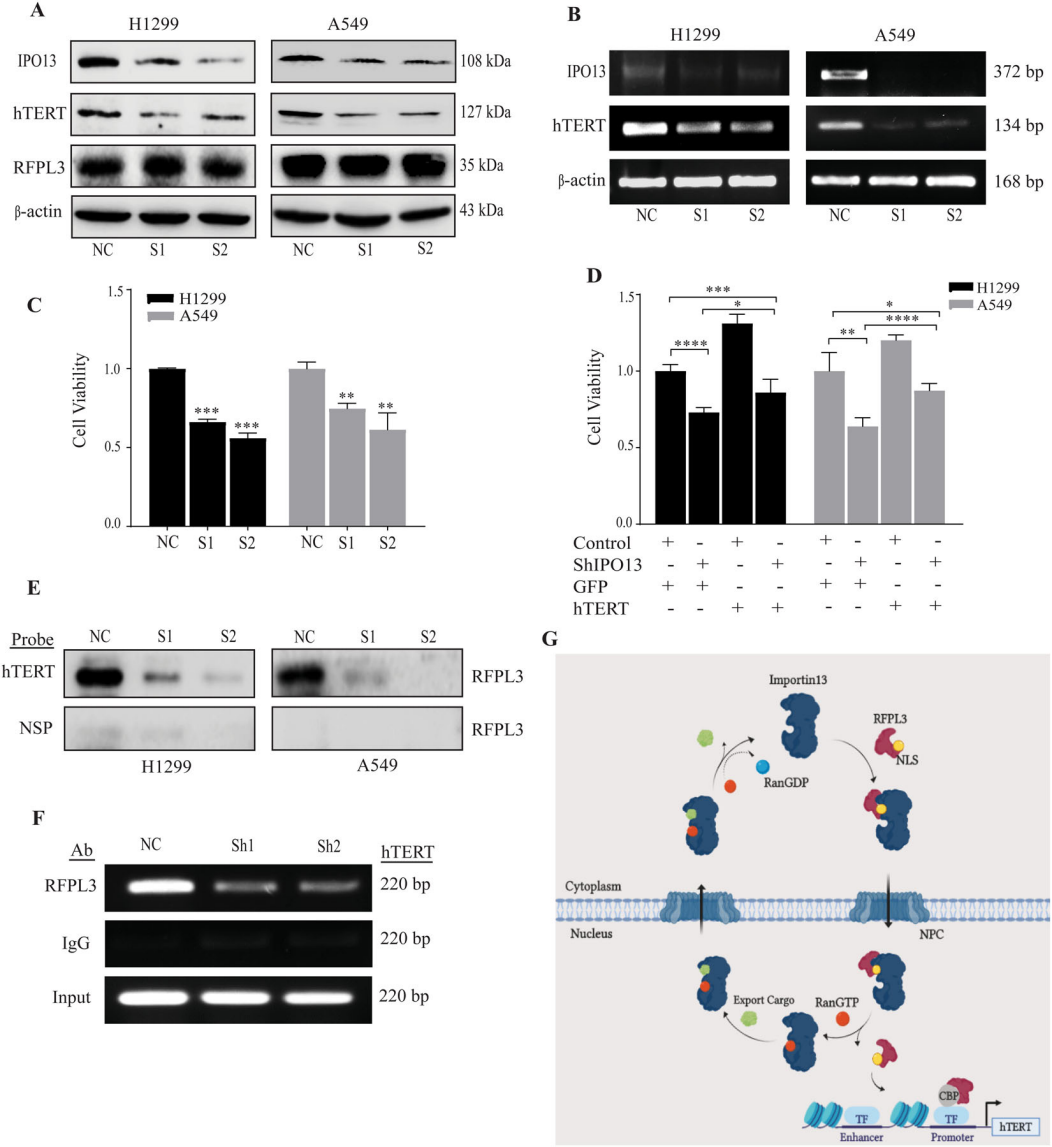
IPO13 implicates in the viability of NSCLCs by downregulation of hTERT. **A**, **B** Downregulation of hTERT expression at protein and mRNA levels in H1299 and A549 cell lines treated with IPO13 siRNA for 48 h. **C** MTT assay used to detect cell viability in (H1299 and A549) cells transfected with IPO13 siRNA (*n* = 3, **P < 0.01, ***P < 0.001). **D** Cell proliferation assay in H1299 and A549 cells upon stable knockdown of IPO13 then overexpresses hTERT (*n* = 3, **P < 0.01, ***P < 0.001, ****P < 0.0001). **E** Pull-down assay conducted in the nuclear extract of H1299 and A549 cells to evaluate the binding of RFPL3 to hTERT promoter (−378 to +60). NSP, nonspecific. **F** The interaction between RFPL3 and hTERT promoter (−160 to +60) in H1299 cells was validated by ChIP assay. **G** A schematic model of the nuclear transport mechanism of RFPL3. Importin 13 mediates the nuclear transport of RFPL3 by recognizing an active NLS at the C terminal. RFPL3 nuclear translocation, in coordination with CBP’s transcriptional co-activation, promotes hTERT transcription. NPC nuclear pore complex, TF transcription factor, CBP CREB-binding protein.

To identify the potential roles of IPO13 in tumor progression, two siRNAs were transfected to knock down IPO13 expression in H1299 and A549 cells. Both have a high knockdown efficiency against IPO13, as shown in Fig. 5A, B. MTT assay was subsequently carried out to assess the IPO13 effect on the proliferation ability of NSCLC. The cell growth in H1299 and A549 was significantly suppressed when IPO13 expression was depleted compared with the negative control (*P* < 0.001, *P* < 0.01), respectively (Fig. 5C). To clarify the role of hTERT in IPO13-mediated NSCLC cell growth, which is transcriptionally regulated by RFPL3 in lung cancer, we first measured hTERT expression at protein and mRNA levels in A549 and H1299 cells. The results showed that IPO13 knockdown led to the downregulation of hTERT, but nearly had no effect on RFPL3 expression levels (Fig. 5A, B). Then cell proliferation assay was performed in H1299 and A549 cells with stable knockdown of IPO13 and simultaneous overexpression of hTERT. As shown in Fig. 5D, the proliferative capacity was significantly reversed by hTERT overexpression. Since IPO13 was identified as a nuclear import of RFPL3, we next indicated the effect of IPO13 knockdown on the binding of RFPL3 to hTERT promoter by pull-down assay (−378 to +60) and ChIP assay (−160 to +60). The results showed the diminished binding ability of RFPL3 to hTERT promoter when IPO13 was knocked down in H1299 and A549 cells by comparison to the control group (NC) (Fig. 5E, F). Collectively, these results confirmed that IPO13 has multiple roles in tumorigenesis of NSCLC, which upregulates hTERT expression by mediating RFPL3 translocation from the cytoplasm to the nucleus, and further induces its binding at hTERT promoter.

### IPO13 enhances tumorigenesis in NSCLC mouse model

**Fig. 6:**
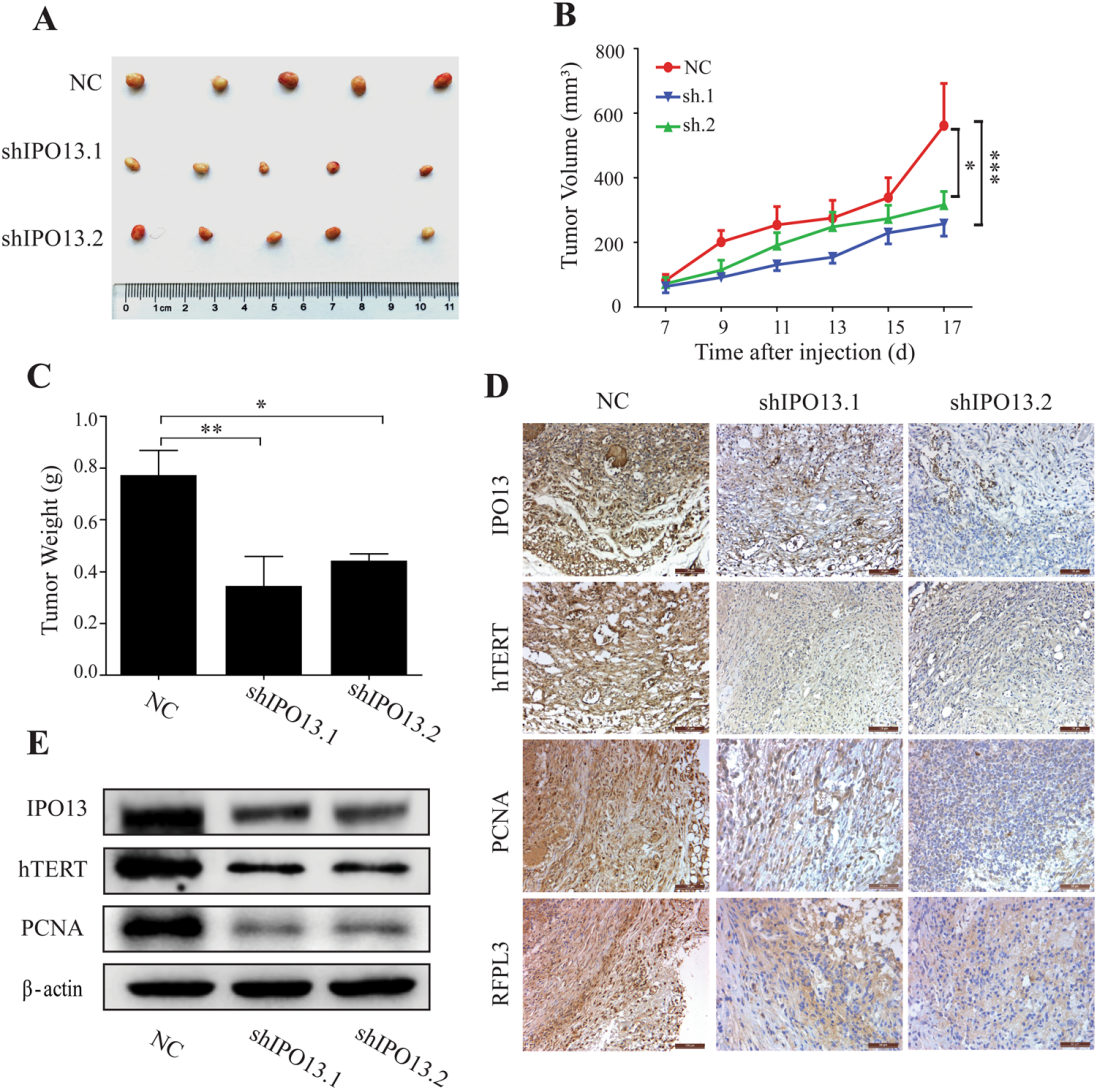
IPO13 enhances the growth of NSCLCs in vivo. **A** The morphology of the xenograft tumor of each nude mouse. **B** The xenograft tumor volume was recorded and calculated by the formula vol = (length × width^2^)/2. **C** Tumor weight of sacrificed mice was measured. *P < 0.05, **P < 0.01, ***P < 0.001. **D** The expression of IPO13, hTERT, PCN, and RFPL3 in tissue sections performed using immunohistochemical (IHC) staining. Scale bar, 100 μm. **E** The expression levels of IPO13, PCNA, and hTERT from tissue lysates detected by western blotting.

To further investigate the effect of IPO13 on tumor growth in vivo, IPO13 was downregulated in the NSCLC mouse model. The A549 cells were transfected with lentivirus particles to generate IPO13-knockdown A549 cells. Then we established a subcutaneous xenograft model in BALB/C nude mice, which we divided randomly to control group (NC), sh-IPO13.1, and sh-IPO13.2 groups with five mice/group. In 30 days after tumor inoculation, mice were sacrificed and the tumor weight was measured (Fig. 6A). Tumors from IPO13-sh cell groups showed slower growth rates than those in the control group NC (*P* < 0.001) (Fig. 6B). Meanwhile, the tumor weight from IPO13-sh groups was higher than the control group (*P* < 0.01) (Fig. 6C). Consistent with in vitro results, immunoblot and IHC staining assays confirmed that IPO13 downregulation significantly suppressed hTERT and PCNA expression levels (Fig. 6D, E). To prove that RFPL3 undergoes nuclear translocation, we stained RFPL3 in mice tissues, where it showed cytoplasmic localization in IPO13-sh groups compared with the control group (nuclear) (Fig. 6D). These findings approved again the oncogenic function of IPO13 in lung cancer progression by mediating the nuclear translocation of RFPL3 that activated the hTERT transcription in vivo and in vitro.

### IPO13 expression is significantly correlated with hTERT expression and EGFR mutations in NSCLC tumor

**Fig. 7:**
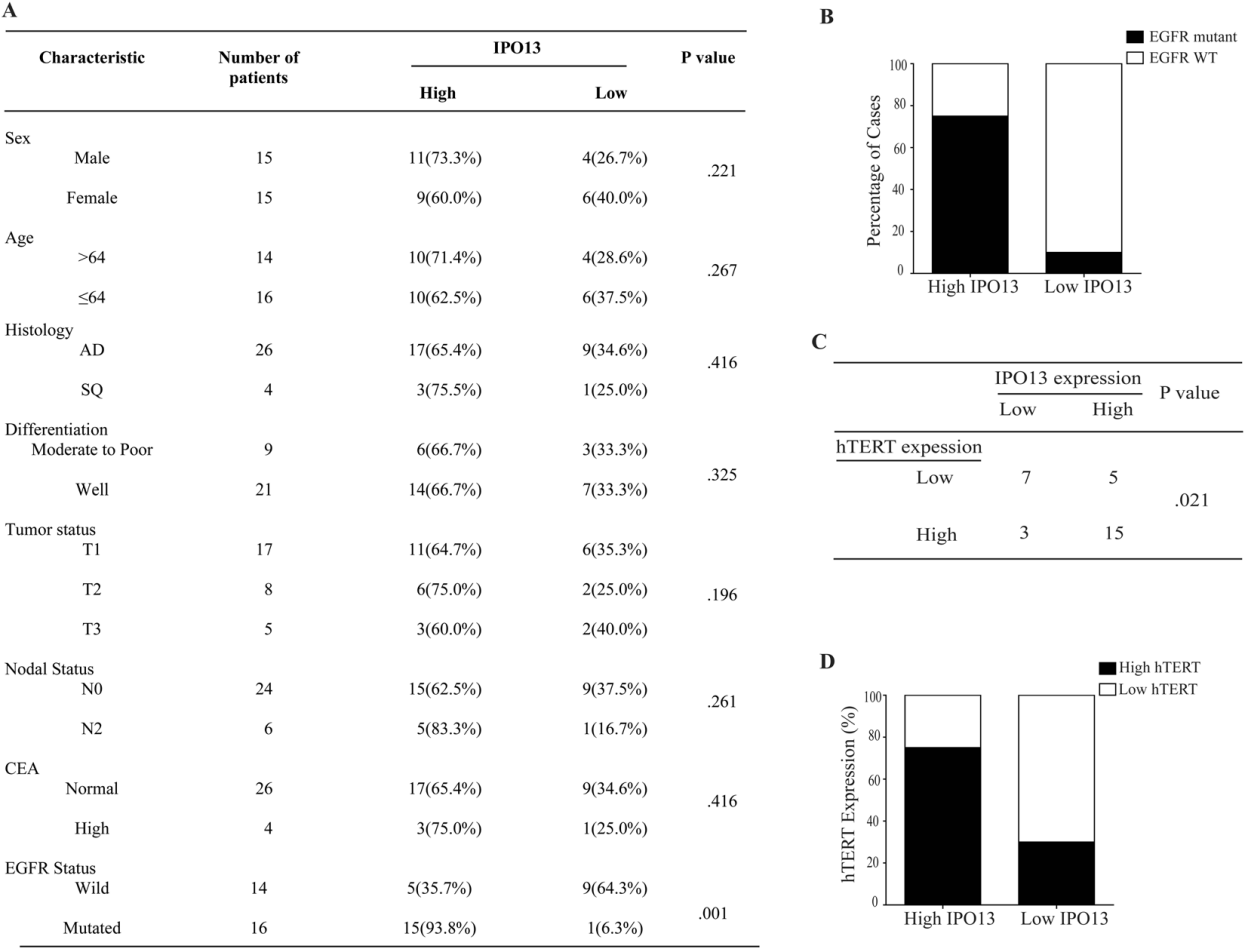
IPO13 overexpression is positively associated with hTERT expression and EGFR status in NSCLC tissues. **A** The association of IPO13 expression level with clinicopathologic features from 30 patients with NSCLC. **B** IPO13 expression level was positively correlated with EGFR mutation status (*P* = 0.001). **C** The correlation between the expression level of IPO13 and hTERT in human lung tissues from 30 patients (*P* < 0.05, χ^2^ tests). **C** The percentage of hTERT expression level in patients with either low or high IPO13 expression.

To evaluate the clinical correlation between IPO13 expression and patient’s clinicopathological characteristics, IHC staining was performed using 30 NSCLC tissue samples. Samples have been divided into two groups based on the protein expression level (high and low) (Fig. 7A). IPO13 upregulation was tightly associated with EGFR mutation status (*P* = 0.001) (Fig. 7A, B). Meanwhile, no significant correlation was detected with sex (*P* = 0.221), age (*P* = 0.267), histology (*P* = 0.416), differentiation (*P* = 0.325), T classification (*P* = 0.196), lymph-node metastasis (*P* = 0.261), and carcinoembryonic antigen CEA (*P* = 0.416) (Fig. 7A). Consistent with the results in vitro and in vivo, Fig. 7C showed that IPO13 expression was positively correlated with hTERT expression in NSCLC patients (*P* < 0.05, χ^2^ tests). In 30 cases tested, 20 cases showed high IPO13 expression; about 75% (15 cases) displayed high expression of both IPO13 and hTERT. While 70% (7 cases) showed low expression of hTERT and IPO13 simultaneously (Fig. 7C).

### EGFR signaling regulates IPO13 expression in EGFR-mutant NSCLC

**Fig. 8:**
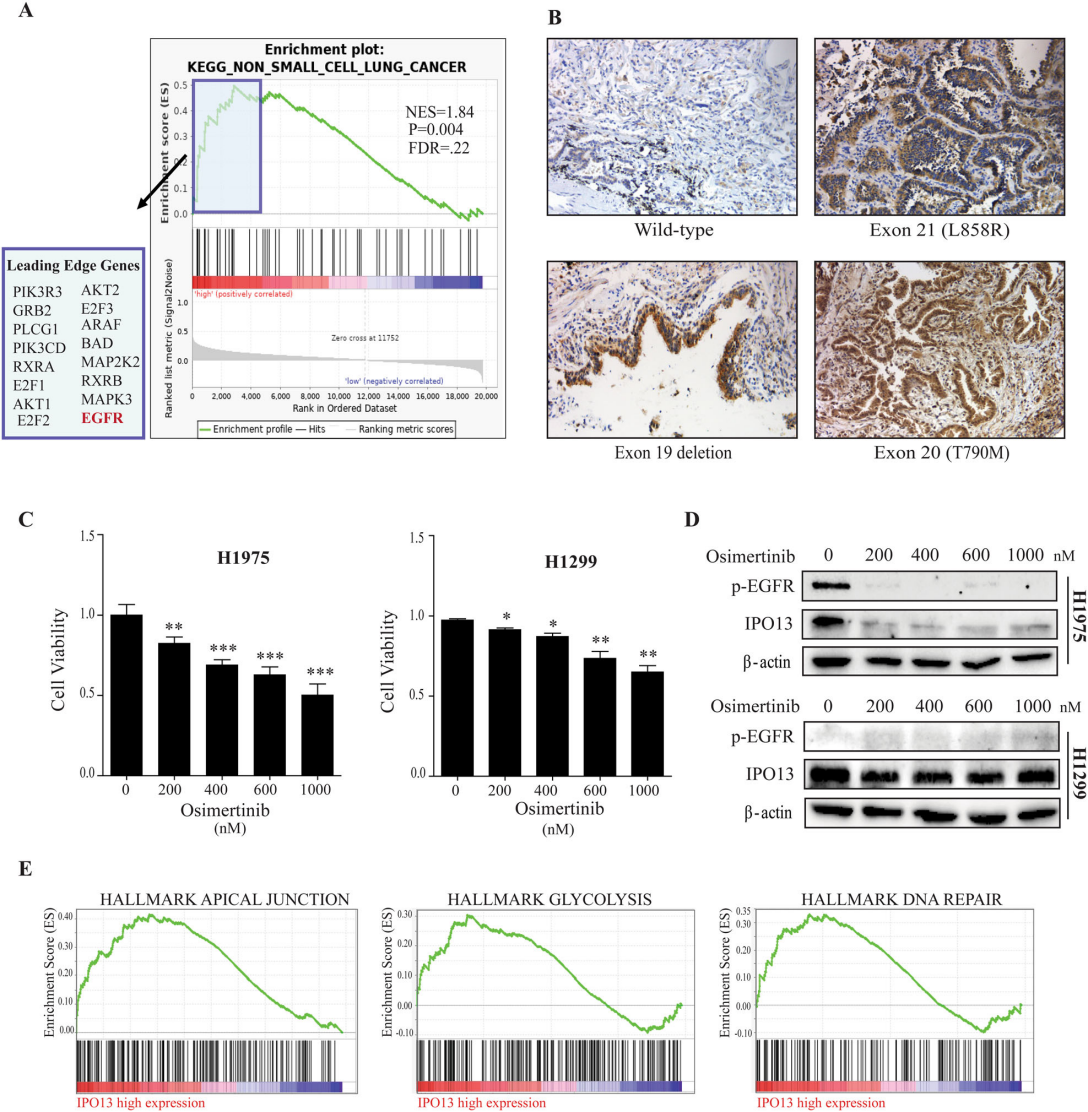
EGFR signaling regulates IPO13 expression in EGFR mutation-positive NSCLC. **A** Gene Set Enrichment Analysis (GSEA) showed genes that are positively correlated to high IPO13 expression on the left side (red), while genes with negative correlation are shown on the right side (blue). **B** IHC analysis of IPO13 expression in NSCLC tumor tissues with various EGFR status (wild and mutant). Scale bar, 100 μm. **C** Human lung cancer cells H1975 (L858R and T790M) and H1299 (wild type) treated with different concentrations of osimertinib for 24 h; then MTT assay was used to test the viability of the cells. Data presented as mean ± S.D. *P < 0.05, **P < 0.01, ***P < 0.001. **D** The expression of IPO13 protein and phosphorylation levels of EGFR in treated cells detected by immunoblot assay. **E** Significant pathways obtained by GSEA that strongly correlated with IPO13 expression, including apical junction, glycolysis, and DNA repair.

According to the positive correlation between IPO13 upregulation and EGFR mutations in NSCLC tumor, GSEA was performed on the gene ontology (GO) and KEGG databases. As Fig. 8A shows, GSEA analysis of the KEGG database confirmed that IPO13 was overexpressed in NSCLC. Also, enrichment analysis showing 16 genes in different signaling pathways (leading-edge genes) is highly correlated to IPO13 upregulation in NSCLC. Notably, EGFR was most highly related with IPO13 upregulation (ES = 0.478). Based on these results, we predict that IPO13 overexpression in NSCLC correlates with EGFR upregulation. To verify this hypothesis, we carried out IHC staining analysis on NSCLC tissues with various EGFR status (Fig. 8B). IPO13 expression levels were significantly higher in NSCLC tissues harboring EGFR mutation compared with those with wild- type EGFR. To observe the oncogenic role of EGFR signaling in regulating IPO13 expression, we studied the effect of Osimertinib, a third generation of EGFR-TKI^29^ on IPO13 abundances in NSCLC cell lines. Treatment of H1299 (wild-type EGFR) and H1975 (L858R, T790M)^30^ with different concentrations of Osimertinib resulted in a significant dose-dependent reduction of cell viability in both cells, as compared with the control group (Fig. 8C). As well, Osimertinib has reversed IPO13 expression and phosphorylation levels of EGFR in H1975. However, no effect on IPO13 expression and EGFR phosphorylation levels in H1299 cells was observed (Fig. 8D). Together, these observations suggest that IPO13 expression is regulated by EGFR signaling in its mutation of NSCLC.

### Significant phenotypes associated with high IPO13 expression

Analysis of predicted phenotypes using GSEA showed that IPO13 was involved in functional pathways, including glycolysis, apical junction, DNA repair (Fig. 8E), myogenesis, E2F targets, and heme metabolism (data not shown).

## Discussion

The accuracy of protein expression levels and subcellular distribution in the cells determines their regular biological functions. As a nuclear protein, RFPL3 can interact with CBP and act as a transcriptional factor of the hTERT promoter in the nucleus to promote hTERT expression^31^, the pathway of which is closely associated with tumorigenesis and progression. Considering that the mechanism that regulates RFPL3 nuclear translocation has not been reported yet, our research provided novel and detailed insights into such mechanisms and the components participating in this mechanism.

Twenty NTRs belonging to the importin β family have been identified, which play a key role in the protein transportation system^32^. In addition to the nucleocytoplasmic transport function, importin-β/karyopherin-β1 showed a regulatory role in mitosis and modulated taxane sensitivity in cancer cells^33^. The specific members of importin-β superfamily, such as importin 13 (IPO13)^34^ and exportin 4 (XPO4)^35^, have a bidirectional transport activity. In this study, we performed profiling of importin-β family members in NSCLC and identified IPO13 as a specifically upregulated KPNB subunit in NSCLC (Supplementary Fig. 1). IPO13 is a poorly studied NTR with a few import and export cargoes. It is reported to regulate nucleocytoplasmic shuttling of transcription factors during lung embryogenesis^36^. However, the relationship between the molecular expression level of IPO13 and its potential function in lung cancer development has not been defined clearly. In our current study, we provide the first evidence that IPO13, as a nucleocytoplasmic shuttling protein, is involved in RFPL3 translocation from the cytoplasm to the nucleus in lung cancer cells. Moreover, combining with our previous study, the accumulation of RFPL3 in the nucleus will lead to the upregulation of hTERT transcription and the further aggravation of the malignant degree of the tumor.

Concerning that RFPL3 belongs to RFPL protein family that has the same structural characteristics with Ret finger proteins (RFP), such as the Ring-finger and B30.2 domains connected by coiled-coil domain, and the coiled-coil domain that has been identified to be responsible for the dot-like structure of RFPL proteins, consistently, our results uncovered the grainy pattern of RFPL3 in the cytoplasm/membrane of NSCLC cells^27,37^. In order to detect which domain of RFPL3 regulates its nuclear localization, further mechanistic studies were performed by using the molecular cloning technology to construct different fragments of RFPL3-FLAG plasmid, and observing the subcellular distribution of RFPL3 after transfecting lung cancer cells with these plasmids. The results showed that amino acid residues (127–235) at the C terminus were critical for RFPL3 nuclear localization (Fig. 4). This finding guides us to speculate the presence of functional NLS in B30.2 domain at the C terminal of RFPL3, which includes PRY and SPRY domains. IP assay further confirmed our speculation that the PRY and SPRY domains within RFPL3 interact with IPO13. Of course, further research is needed to identify the NLS location more precisely in RFPL3. More significantly, the knockdown of IPO13 resulted in pronounced inhibition of NSCLC cell viability through hTERT downregulation in vitro (Fig. 5). Correspondingly, in vivo experiments again demonstrated that IPO13 promotes the progression of lung cancer by mediating RFPL3’s nuclear translocation and upregulation of hTERT expression (Fig. 6). Thus, IPO13 and its nuclear transport functions might provide a vital strategy in NSCLC treatment. Based on our previous studies and our current findings, a model of RFPL3’s translocation from the cytoplasm to nuclear localization is generated (Fig. 5G).

Epidermal growth factor receptors (EGFRs) are 170-kilodalton transmembrane glycoproteins, belonging to receptor tyrosine kinase (RTK) family. The binding of EGF or other ligands to EGFRs leads to phosphorylation of the intracellular portion (tyrosine kinase domain) that results in the activation of various signaling cascades^38,39^. EGFR mutations have been detected in 43–89% of NSCLC patients^40^, and the most common mutations were exon 19 in-frame deletion and missense mutation L858R in exon 21^41^. Many clinical trials showed a marked anticancer response to EGFR tyrosine kinase inhibitors (EGFR-TKIs) in EGFR-mutant NSCLC cases^42–44^. However, the acquired resistance to EGFR-TK inhibitor therapies forms the main clinical therapeutic barrier. T790M is a secondary mutation in the EGFR kinase domain (exon 20) that is identified to be the primary resistance mechanism for EGFR tyrosine kinase inhibitor, which is detected in approximately 60% of EGFR-TKI resistance in NSCLC cases^45,46^. Our current data revealed that the presence of EGFR mutation was significantly related to overexpression of IPO13. Furthermore, suppression of EGFR signaling by Osimertinib (Tagrisso, AZD9291), a third-generation-irreversible EGFR-TK inhibitor with a high affinity for EGFR-mutant protein^47,48^, resulted in the inhibition of IPO13 expression in the EGFR-mutant NSCLC cell line, H1975 cell line, which harbors L858R mutation (exon 21) and T790M mutation (exon 20)^30^. In contrast, no effect was detected in cells expressing wild-type EGFR, indicating that the abundance of IPO13 was probably depending on EGFR signaling in NSCLC (Fig. 8). Hence, our results have suggested IPO13 as a potential target of anticancer drugs to overcome the acquired EGFR-TKI resistance in EGFR-mutant NSCLC. The underlying molecular mechanism for the regulation of IPO13 expression in NSCLC with EGFR mutation will be further explored and clarified in our future study.

In summary, our paper exposes a novel pathway by which IPO13 facilitates the nuclear entry of RFPL3, which acts as a specific transcription factor of hTERT to control its expression and to be further involved in lung cancer development. Thus, the identification of such molecular mechanism involved in the subcellular distribution of RFPL3 in lung cancer cells might provide a new potential target for anticancer therapies. It also provides the possibility of the combined strategy for NSCLC treatment based on hTERT-targeted therapy. In conclusion, IPO13’s nuclear transportation function is a crucial point in NSCLC progression, and the revelation of these functions might help us to discover novel therapeutic strategies.

## Supplementary information

supplemetary figure and table legends

Supplemental figure 1

Table1
