# Supplementary material for: Importin 13 promotes NSCLC progression by mediating RFPL3 nuclear translocation and hTERT expression upregulation
Source: Cell Death Dis. 2020 Oct 20;11(10):879. doi: 10.1038/s41419-020-03101-9 (PMC7575581; doi:10.1038/s41419-020-03101-9)
Supplement: Supplementary file 1 — supplemetary figure and table legends [file 41419_2020_3101_MOESM1_ESM.docx]

**Supplementary figure and table legends:**

**Supplemental figure1.** **Profiling of** **importin β family members alteration in NSCLC.** (A-E) Analysis of importin β family members expression levels across cancer/normal lung from Oncomine database. ADC: lung adenocarcinoma. SCLC: small cell lung cancer. CAR: lung carcinoid tumor. SCC: squamous cell lung carcinoma. (F) RT-PCR performed to examine the mRNA expression levels of KNPB family members in normal epithelial lung cell HBE, and NSCLC cell lines (A549, H1299, H1975).

**Table1. List of primers used in this study.**
