# Supplementary material for: Importin 13 promotes NSCLC progression by mediating RFPL3 nuclear translocation and hTERT expression upregulation
Source: Cell Death Dis. 2020 Oct 20;11(10):879. doi: 10.1038/s41419-020-03101-9 (PMC7575581; doi:10.1038/s41419-020-03101-9)
Supplement: Supplementary file 3 — Table1 [file 41419_2020_3101_MOESM3_ESM.docx]

Table1. List of primers used in this study.

| Gene name | Primer sequence |
| --- | --- |
| IPO13 | 5’-CGCTGCACCAGCTCTACTATG-3’ |
| IPO13 | 5’-CAGCCTGGAAGAGTCGTACC-3’ |
| KPNB1 | 5’-TTGGAGGGAGGAAGTAAGGGAA-3’ |
| KPNB1 | 5’-ACTGTGAGGCAGAACTAGGC-3’ |
| XPO7 | 5’-GGCAGCTACATGCCTTACCA-3’ |
| XPO7 | 5’-GGGTGGTGTCTGCTTGATTA-3’ |
| TNPO2 | 5’-CGGAATCCCAACTGCTCCTT-3’ |
| TNPO2 | 5’-GGCACCGTTTTTCCTTCTCG-3’ |
| IPO11 | 5’-GGTGTGAAACTGCTGGGTCAT-3’ |
| IPO11 | 5’-AGTATCCTGACTGGTGGCCT-3’ |
| XPO1 | 5’-GGGCTGAAAACTCAACCGAG-3’ |
| XPO1 | 5’-GCAGGAGTAGGGGGTGTAGA-3’ |
